# The “16‐gram window” of contact‐force: A new criterion for very high‐power short‐duration ablation

**DOI:** 10.1002/joa3.70076

**Published:** 2025-05-06

**Authors:** Dimitrios Bismpos, Jan Wintrich, Valerie Pavlicek, Raphael Spittler, Alexander P. Benz, Michael Böhm, German Fernandez Ferro, Felix Mahfoud, Thomas Rostock, Christian Ukena

**Affiliations:** ^1^ Department of Internal Medicine III, Cardiology, Angiology and Intensive Care Medicine, University Hospital Saarland University Homburg Saar Germany; ^2^ Department of Cardiology/Angiology, Marien Hospital Herne Ruhr University Herne Germany; ^3^ Department of Cardiology II/Electrophysiology, Center for Cardiology University Hospital Mainz Mainz Germany; ^4^ Department of Cardiology, University Heart Center University Hospital Basel Basel Switzerland; ^5^ Cardiovascular Research Institute Basel (CRIB), University Heart Center University Hospital Basel Basel Switzerland

**Keywords:** atrial fibrillation, contact‐force sensing ablation, pulmonary vein isolation, radiofrequency ablation, very high‐power short‐duration ablation

## Abstract

**Background:**

Very high‐power short‐duration (vHPSD) ablation with the novel QDOT™ catheter allows the regulation of target temperature by automatically adjusting flow and power during a 4 s application of 90 W. However, the optimal contact force for sufficient lesion creation is unknown.

**Methods:**

We enrolled 73 patients with symptomatic atrial fibrillation undergoing pulmonary vein isolation (PVI) using the QDOT catheter in the vHPSD mode (90 W, 4 s). Ablation metrics associated with suboptimal applications, defined as either an impedance drop of ≤5% or a cumulative temperature‐limited energy ≤330 J, were collected and analyzed.

**Results:**

A total of 3881 vHPSD applications (53.2 applications per patient) with a mean contact force (CF) of 12.8 ± 6.6 g were analyzed. Significant CF variability and intermittent loss of contact were documented in 18.2% and 8.8% of the applications, respectively. A ΔImp ≤ 5% occurred in 3.9% of vHPSD applications, while a cumulative energy ≤ 330 J was observed in 3% of the applications. Applications with a mean CF < 6 g and >22 g were associated with an inadequate impedance drop (10.3%, Phi coefficient 0.118, *p* < .001) and total applied energy (7.8%, Phi coefficient 0.094, *p* < .001) respectively. At superior PV segments with thick atrial walls, significantly more applications with cumulative energy ≤330 J (4.2% vs. 2.5%; *p* = .007) were observed, especially when mean CF > 18 g was applied (8.4%, Phi coefficient 0.093, *p* = .003).

**Conclusion:**

A lower but also a higher mean contact‐force was associated with suboptimal vHPSD applications. Hence, a “16‐gram window” of contact‐force, from 6 to 22 g, could optimize energy application in vHPSD ablation.

## Graphical abstract

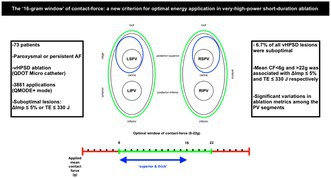

## 1 INTRODUCTION

In the modern era of catheter ablation of atrial fibrillation (AF), arrhythmia recurrence remains a significant clinical challenge, particularly due to electrical pulmonary vein (PV) reconnection.^1^ In recent years, a variety of ablation methods using different sources of energy have been developed, though radiofrequency (RF) and single‐shot devices like Cryoballoon (CB) remain the most commonly applied energy sources.^2^ While single‐shot devices and point‐by‐point RF ablation catheters have shown comparable acute‐ and long‐term outcomes, the latter provides superior adaptability to different PV anatomies.^3^

To improve procedural efficiency and safety as well as long‐term effectiveness, a contact force (CF)‐sensing RF technology aiming for “very high‐power short‐duration (vHPSD)” ablation has been recently developed. The novel CF‐sensing QDOT™ catheter (Biosense Webster Inc., CA) uses a power of 90 W for a duration of 4 s to increase the resistive heating phase responsible for tissue necrosis while minimizing conductive heating, thus sparing adjacent tissues.^4^, ^5^, ^6^ This results in wider, shallower, more homogeneous and well‐defined lesions.^4^, ^7^, ^8^ Furthermore, the intensity of a transient hemorrhage ring at the periphery of the lesion representing a potential mechanism of PV reconnection may be decreased.^7^ These effects are achieved through modulation of power in order to maintain a target temperature (maximum 60°C) during the vHPSD applications (QMODE+ modality), using real‐time, optimized temperature monitoring assessed by microelectrodes and six superficial thermocouples.^4^, ^5^

Several advances like contact‐force and lesion distance measurement as well as ablation index‐guided (AI) lesion generation can improve lesion quality and thereby positively impact procedural safety and long‐term outcomes.^9^ However, no surrogate parameters indicating a sufficient lesion generation for vHPSD ablation are currently available. The present study aimed at investigating procedural parameters and optimal contact‐force profiles for sufficient lesion generation with vHPSD.

## 2 METHODS

### 2.1 Study subjects and design

This prospective, two‐center study enrolled patients with symptomatic paroxysmal or persistent AF undergoing PV isolation (PVI) for the first time between September 2022 and February 2023. The vHPSD approach was selected for the ablation using the QDOT Micro catheter (Biosense Webster). Ablation metrics were recorded during the procedure. All patients gave written informed consent. The study was approved by the local institutional review board and was based on the ethical guidelines of the Declaration of Helsinki.

### 2.2 Pulmonary vein isolation procedure

After transseptal puncture, electro‐anatomical mapping of the left atrium was performed with the guidance of the CARTO three‐dimensional mapping system (Biosense Webster). The QDOT Micro catheter in vHPSD mode (Qmode +) using 90 W for 4 s with a maximum temperature of 60°C was used for point‐by‐point ablation for all applications (anterior and posterior). An interlesion distance of 4–6 mm was required. The procedural endpoint was defined as a bidirectional conduction block between the left atrium and the pulmonary vein. If the PVs were not isolated after first‐pass circumferential ablation or a segmental reconnection after a waiting period of 20 min was documented, additional ablation with vHPSD or 50‐W mode with an AI of 350–450 was performed. In patients with common‐type atrial flutter, an ablation of the cavotricuspid isthmus (CTI) was performed with the 50‐W mode and an AI of 450–550. Organized atrial tachycardia occurring after PVI was targeted for additional ablation at the discretion of the operator.

### 2.3 Definition of suboptimal ablation lesions

Significant CF variability was defined as CFvar60% >10 g; i.e. intermittent catheter contact expressed by a high variability in the middle range of CF values.^10^ Intermittent loss of contact was defined as the lowest quintile of CF <3 g.^11^ Applications were defined as incomplete if the duration was limited to <3.7 s due to temperature rise and were excluded from further analysis. Suboptimal vHPSD applications were defined as either an impedance drop of ≤5% or a cumulative temperature‐limited applied energy of ≤330 J. The cut‐offs were chosen based on the respective 5th percentile. Based on the ablation algorithm of the QMODE+ mode, a temperature ≥60°C results in a downregulation of power output during the four‐second application. Therefore, assessment of the total applied energy appeared a more appropriate surrogate than change of temperature or absolute temperature. Due to the unique nature and recent development of the vHPSD ablation, data linking total applied energy to lesion formation are currently lacking; however, its evaluation as a surrogate parameter is increasingly used in clinical practice and is endorsed by the vendor (Biosense Webster).

### 2.4 Endpoints

The aim of this analysis was to describe the optimal contact‐force profile for a sufficient lesion generation using the QDOT Micro catheter in vHPSD Mode (90‐W, 4 s). Ablation metrics, including applied contact‐force values associated with suboptimal ablation lesions, were collected and analyzed across the PV segments.

### 2.5 Statistical analysis

Data are presented as mean ± standard deviation (SD) or number (percentage) unless otherwise specified. Continuous variables were tested for normal distribution with the Kolmogorov Smirnov test and by visual inspection of quantile plots. Continuous variables between two groups were compared with the Wilcoxon rank‐sum test, while the Kruskal–Wallis test was used for comparing continuous variables between more than two groups. Comparisons for categorical variables were performed with the Pearson Chi‐Square test. To compare categorical variables between more than two groups, results were corrected according to Bonferroni. The Spearman *ρ* correlation coefficient was used to assess the correlation between variables. The Phi coefficient was used to assess the association between two binary variables. A two‐tailed *p* value of <.05 was regarded as statistically significant. All statistical analyses were performed with SPSS statistical software (version 29.0, SPSS Inc., Chicago, Illinois). All authors had full access to the data and take full responsibility for their integrity.

## 3 RESULTS

### 3.1 Mean ablation metrics

A total of 4067 ablation applications with the QMODE+ mode were performed among 73 study patients who underwent first‐time PVI. A total of 186 applications were regarded as incomplete (duration <3.7 s), 3,881 applications were included in the analysis, which corresponds to 53.2 applications per patient. Mean contact force (CF) was 12.8 ± 6.6 g. Contact‐force variability, defined as the difference between maximum and minimum CF, was 17.8 ± 11 g. Significant CF variability was evident in 18.2% of applications, while intermittent loss of contact occurred in 8.8% of all applications.

### 3.2 Characteristics of suboptimal lesions

**FIGURE 1:**
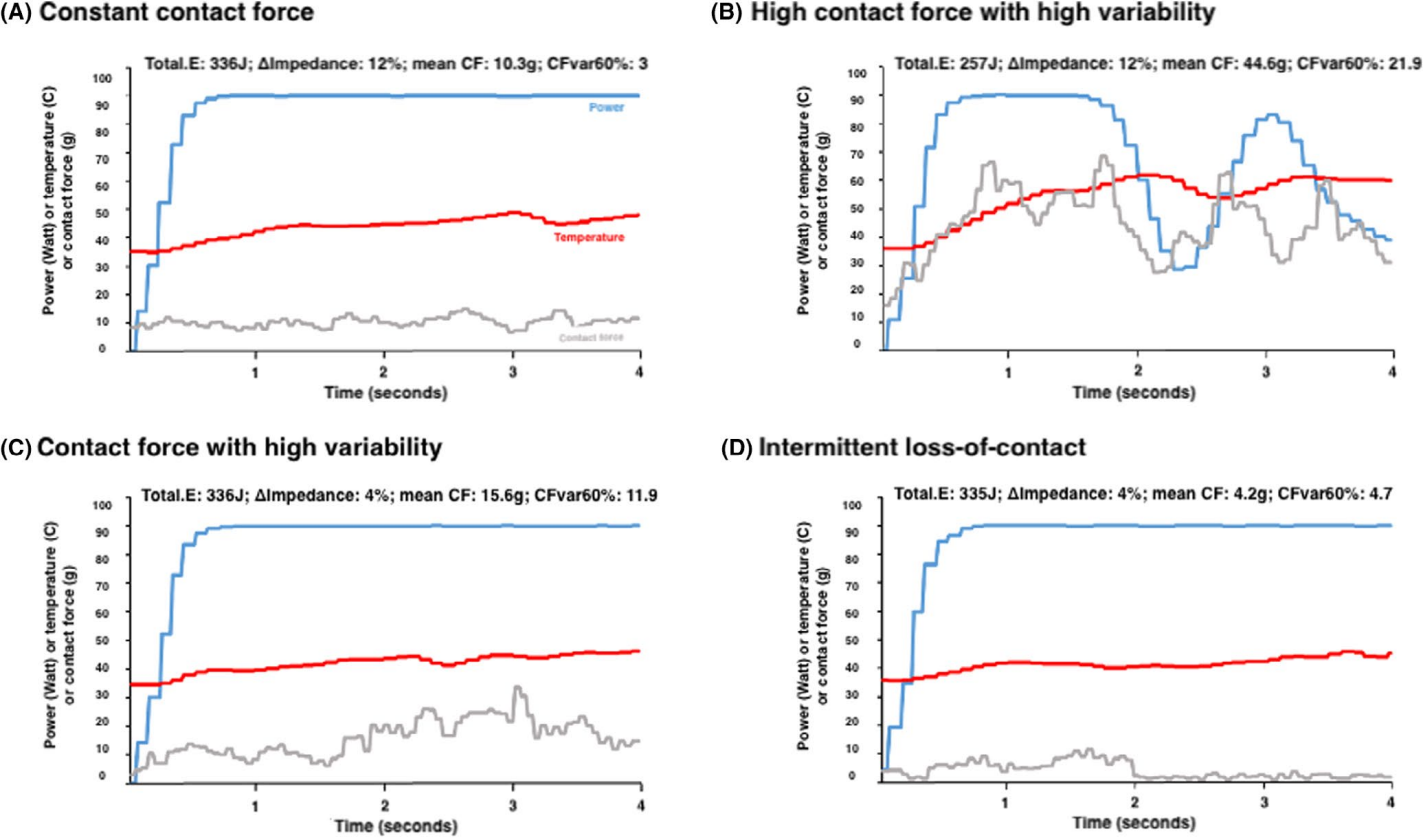
Examples of vHPSD applications.°C, degrees Celsius; CFvar60%, significant contact‐force variability; g, grams; J, Joule; mean CF, mean contact force; TotalE, total applied energy; vHPSD, very high‐power short duration; ΔImpedance, impedance drop.

Varying examples of suboptimal lesions are presented in Figure 1. Mean impedance drop (Δimpedance) was 10.1 ± 3.1%, while 3.9% of all vHPSD applications were associated with an impedance drop ≤5%, corresponding to a mean impedance drop of 4.5 Ohm (Table S1). Applications with Δimpedance ≤5% had a significantly lower maximum temperature (46.4 ± 4.3 vs. 48.9 ± 3.9°C; *p* < .001) and lower temperature rise (11 ± 4.2 vs. 14.1 ± 3.8°C; *p* < .001). However, the total applied energy of these applications did not differ significantly (333 ± 16 vs. 334 ± 8 J; *p* = .131). Applications with Δimpedance ≤5% had a significantly lower mean CF (9.9 ± 6.5 vs. 12.9 ± 6.5 g; *p* < .001) and higher rates of intermittent loss of contact (22.7% vs. 8.3%; *p* < .001).

The mean cumulative energy was 334.3 ± 8.4 J, while 3% of all vHPSD applications had a cumulative energy of <330 J, corresponding to a mean cumulative energy of 308 J (Table S1). Low cumulative energy vHPSD applications (≤330 J) were characterized by a higher maximum temperature (55.5 ± 6.2 vs. 48.6 ± 3.7°C; *p* < .001) as well as a higher temperature rise (20 ± 6 vs. 13.8 ± 3.6°C; *p* < .001). Moreover, these vHPSD applications demonstrated a higher mean CF (17.6 ± 9.0 vs. 12.6 ± 6.4 g; *p* < .001) and higher rates of significant CF variability (34.2 vs. 17.8%; *p* < .001).

The mean CF correlated with impedance drop (Spearman's rho 0.243, *p* < .001) and cumulative energy (Spearman's rho −0.088, *p* < .001). Rates of suboptimal lesion generation, defined as either Δimpedance ≤5% or total energy ≤330 J, were analyzed across the deciles of CF (Figure S1). Applications with an Δimpedance ≤5% were most frequently observed in the lowest CF decile (10.3%), which corresponded to a mean CF <6 g. Hence, applications with a mean CF <6 g were significantly associated with a Δimpedance ≤5% (Phi coefficient 0.118, *p* < .001). On the other side of the spectrum, applications with total energy ≤330 J were most frequently observed in the highest CF decile (7.8%), corresponding to a mean CF >22 g. Likewise, applications with a mean CF > 22 g were associated with total energy ≤330 J (Phi coefficient 0.094, *p* < .001) (Figure S2A,B).

**FIGURE 2:**
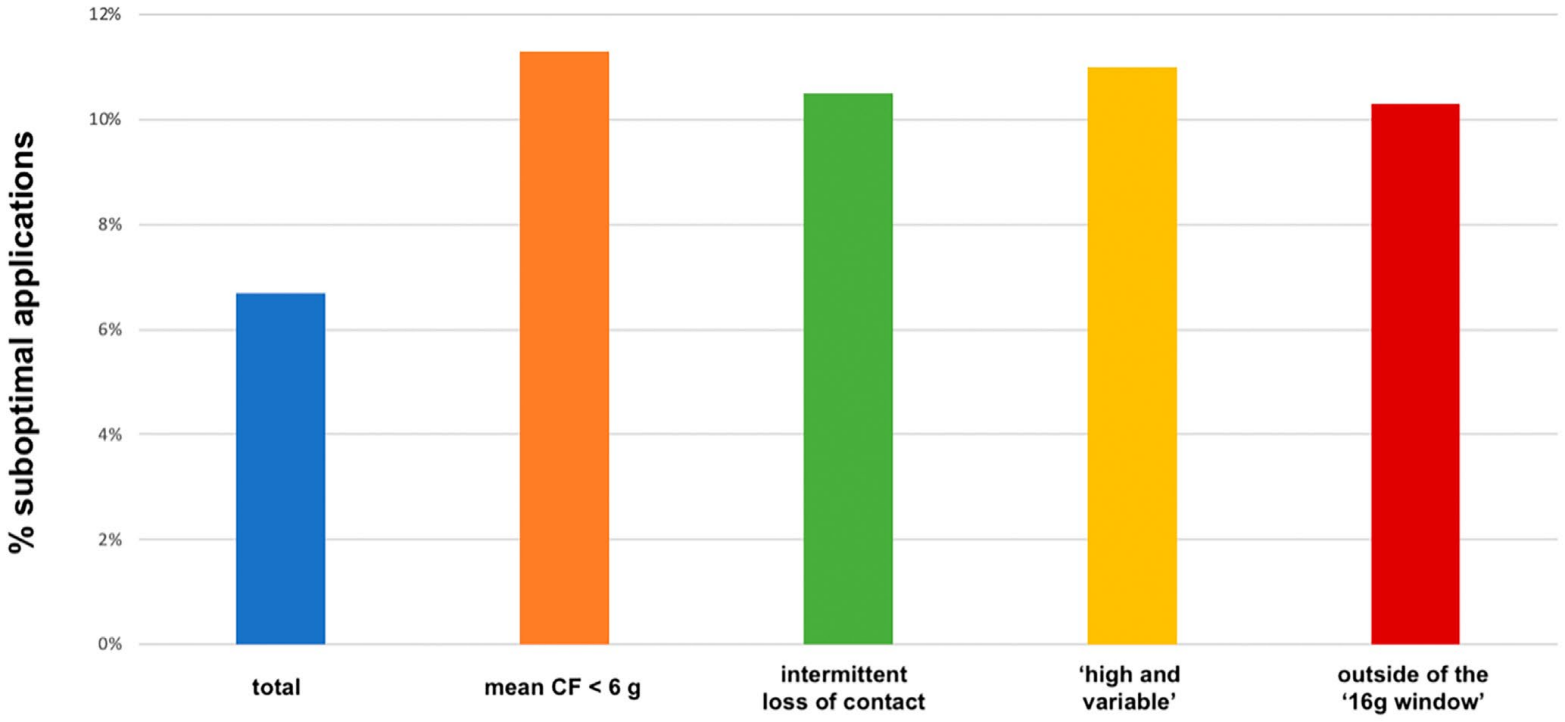
Rates of suboptimal applications (ΔImpedance ≤ 5% or total energy ≤330 J) related to the profile of contact force (CF). CF, contact force; g, grams.

Suboptimal lesion generation occurred in 6.7% of all vHPSD ablations. High rates of suboptimal lesions were most often observed in applications with (i) mean CF <6 g (11.3%), (ii) “high and variable” lesions (defined as a mean CF > 22 g and significant CF variability: 11.0%), (iii) intermittent loss of contact (10.5%), and (iv) lesions outside of the 16 g window (defined as mean CF between 6 and 22 g: 10.3%) (Figure 2).

### 3.3 Differences among the PV segments

A total of 1914 RF applications were performed in the left PVs and 1799 in the right PVs. Ablation metrics were different between the right and left PVs (Table S2).

**FIGURE 3:**
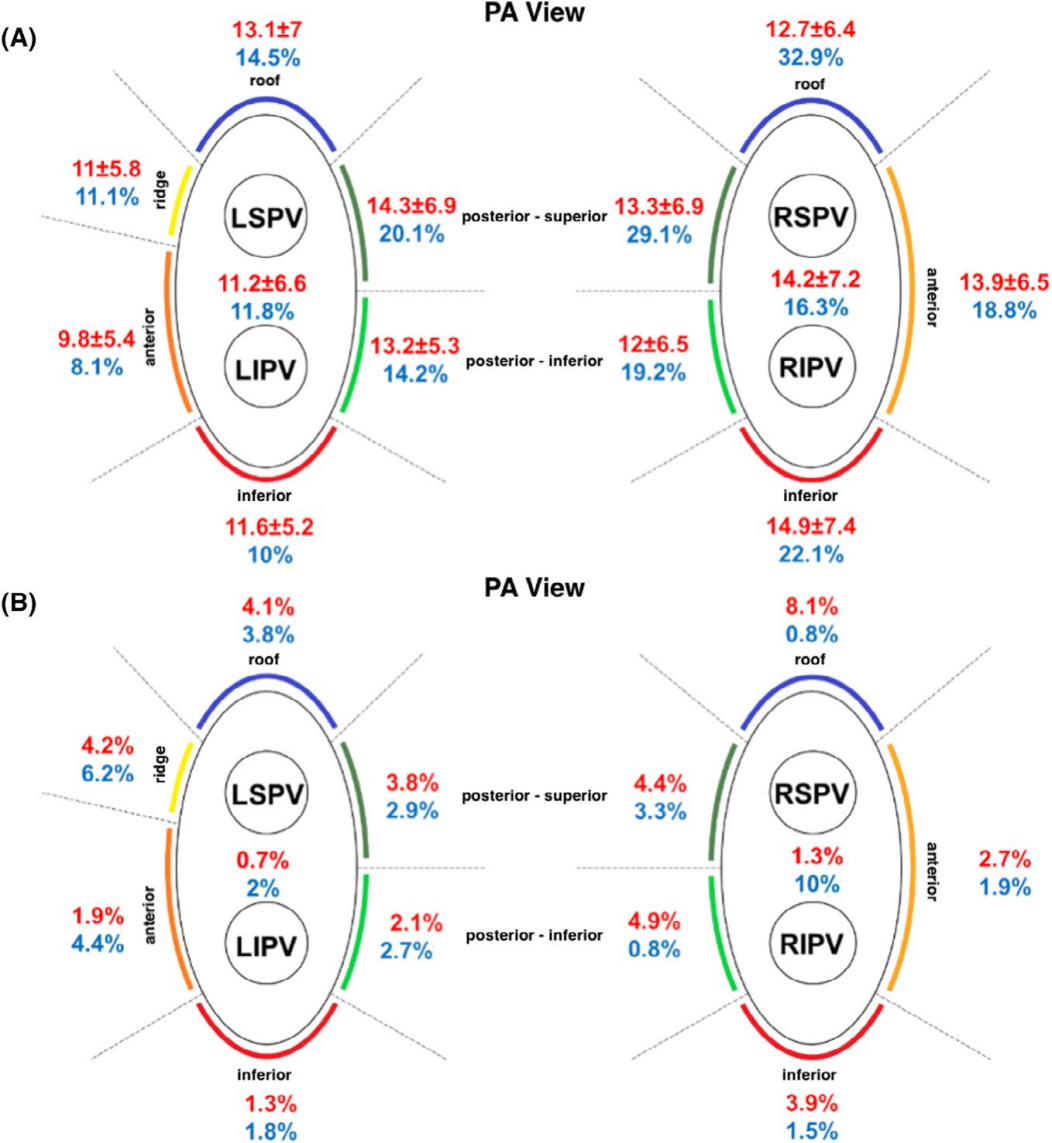
(A, B) Distribution of contact force and suboptimal applications related to the pulmonary vein segments. Normally distributed values are reported as mean ± standard deviation (SD). CFvar60%, significant contact‐force variability; g, grams; J, Joule; LIPV, left inferior pulmonary vein; LSPV, left superior pulmonary vein; PA view, posteroanterior view; PV, pulmonary vein; RIPV, right inferior pulmonary vein; RSPV, right superior pulmonary vein; ΔImpedance, impedance drop.

Mean CF and significant CF variability varied notably among the PV segments (Figure 3A). Localized rates of applications with Δimpedance ≤ 5% as well as total applied energy ≤330 J are presented in Figure 3B. Significant differences were observed for applications with Δimpedance ≤5% and total energy ≤330 J at the given PV segments (*p* = .002). In superior PV segments with thick atrial walls (ridge, roof, carina), a significantly higher number of applications with Δimpedance ≤ 5% (4.7% vs. 3.6%, *p* = .121) and total energy ≤ 330 J (4.2% vs. 2.5%, *p* = .007) were observed.

The spatial distribution of suboptimal applications was analyzed across the CF deciles. In superior PV segments with thick atrial walls, a high number of applications with total energy ≤330 J was observed in the highest CF decile (11.9%), corresponding to a mean CF >22 g (Phi coefficient 0.116, *p* < .001). However, this observation was relevant even in the 8th decile (8.4%), corresponding to a mean CF > 18 g (Phi coefficient 0.093, *p* = .003) (Figure S3A,B).

## 4 DISCUSSION

Herein, ablation metrics associated with insufficient lesion generation using the QDOT Micro catheter in vHPSD Mode (90 W, 4 s) were collected and analyzed. The present study provides the following new information on very high‐power short‐duration ablation: First, suboptimal lesion generation, defined as Δimpedance ≤5% or total energy ≤330 J, occurred in 6.7% of RF applications. Second, significant CF variability and intermittent loss of contact are frequently observed. Third, a significant association between RF applications with a mean CF <6 g as well as CF > 22 g and suboptimal lesions was observed. Finally, suboptimal ablation lesions are nonuniformly distributed to the given PV segments due to varying CF profiles.

The incorporation of CF‐sensing catheters in RF ablation has improved procedural outcomes of AF ablation, particularly when CF stability is achieved.^10^, ^12^ Despite recent technological advancements, catheter‐tissue contact stability remains vital for successful lesion creation, as a balance between sufficient tissue contact and adjacent tissue overheating is needed.^13^, ^14^ Variable or intermittent contact, typically due to ventricular contraction, can be associated with sudden changes in CF values.^10^ Furthermore, respiration under general anesthesia contributes to variable or intermittent contact, which could be attenuated during phases of apnea.^10^, ^15^ In theory, ablations with higher energy can mitigate the negative effects of CF instability, as lesion creation is achieved in a very short duration of time.^16^, ^17^ In this study, mean contact force was 12.8 ± 6.6 g, while intermittent loss of contact (8.8%) and significant CF variability (18.2%) were frequently observed. Therefore, maintenance of optimal contact force should be actively pursued, even during the short duration of vHPSD ablation.^5^ It has already been demonstrated in ablation with lower RF energy and longer duration settings that a minimum CF is needed to achieve stable contact with the tissue and avoid intermittent loss of contact.^11^

Furthermore, it has been reported that generator impedance drop during RF ablation is predictive of lesion size and acute PV reconnection.^18^ Of interest, impedance drop correlates to applied contact force.^19^, ^20^ The present study demonstrates that suboptimal RF applications (ΔImpedance ≤ 5%, 5th percentile) were more likely to have a lower mean CF (9.9 ± 6.5 vs. 12.9 ± 6.5 g; *p* < .001) and higher rates of intermittent loss of contact (22.7% vs. 8.3%; *p* < .001). A minimum CF during ablation appears to be required to achieve a sufficient impedance drop, whereas a further increase in CF will not result in better lesion matrix.^21^ This dynamic seems to apply to vHPSD ablation, as a CF of 8 g is reported to be the optimal cutoff value for adequate impedance drop.^22^ These findings are supported by our data, as applications with a mean CF <6 g were significantly associated with a Δimpedance ≤5% (10.3% vs. 3.1%, Phi coefficient 0.118, *p* < .001).

Interestingly, an increase of CF values during vHPSD ablation leads to lower cumulative applied energy due to the unique temperature‐guided downregulation of power.^23^, ^24^ Of note, experimental studies demonstrated that lesion size does not significantly increase beyond a CF of 15–20 g.^17^, ^24^ The data of the present study are in line with these results, as vHPSD applications with a mean CF > 22 g were associated with total applied energy ≤330 J (7.8% vs. 2.4%, Phi coefficient 0.094, *p* < .001). While this strategy is associated with a lower incidence of steam‐pops,^7^, ^8^, ^12^ it is currently less clear if the downregulation of energy at higher CF levels may have a negative impact on procedural success and clinical efficiency.

In the present study, we observed significant variations of CF distribution among the PV segments. Application rates with ΔImpedance ≤ 5% were higher in the right PVs, particularly in the posterior and roof segments. Contrariwise, cumulative energy ≤330 J was documented more commonly in the left PVs, particularly in the anterior, ridge, and roof segments. Thus, variations in ablation metrics across the PV segments indicate that the diverse anatomic characteristics of the atrial tissue require an anatomically tailored approach to ablation. Areas of the left atrium with thicker walls such as the ridge, roof, and carina may not be suitable for vHPSD ablation.^6^, ^8^, ^12^, ^25^ This finding could be supported by our data, as superior PV segments with thick atrial walls (ridge, roof, carina) were more likely to have suboptimal applications due to a higher number of applications with insufficient cumulative energy. The reason for the diminished effect of vHPSD ablation on these locations could be the broad but shallower lesions achieved during energy application,^8^ as well as insufficient CF stability. Therefore, careful monitoring of CF values when isolating these PV segments is warranted. Alternatively, a change to conventional‐power temperature‐controlled mode (CPTC) may be required.^4^, ^6^, ^14^, ^26^ Of note, in the recently published QDOT‐by‐LAWT trial,^27^ the combination of vHPSD with standard‐power ablation guided by LA wall thickness was not inferior to the CLOSE protocol. On the other hand, the shallow lesions of vHPSD may be more suitable for left atrial tissue proximal to the esophagus, such as the posterior wall of the left PV, thus minimizing injury to the esophagus.^28^ The versatility of the QDOT catheter, which allows operators to seamlessly switch between vHPSD and CPTC, could therefore provide operators the flexibility to adjust to various LA anatomies.^6^

Several advances in contact‐sensing and lesion distance measurement have led to the development of indexes to improve procedural efficacy and safety, like lesion size index (LSI)^29^ and the use of local impedance drop to predict lesion characteristics.^30^ However, these indexes as well as the optimal contact‐force profile for a sufficient lesion generation in vHPSD ablation have not been verified thus far. We demonstrate that applications with a mean CF <6 g and >22 g were associated with insufficient impedance drop and low cumulative applied energy, respectively. Therefore, we propose the use of a “16‐gram window” (6–22 g) of contact‐force for optimized vHPSD applications. It is important to note, that though significant (*p* < .001), a rather moderate statistical association was documented between mean CF and insufficient impedance drop (Spearman's rho 0.243) as well as cumulative applied energy (Spearman's rho −0.088). Nevertheless, considering the vast complexity of AF ablation, these findings indicate that the use of a “16‐gram window” of contact‐force could contribute to optimized vHPSD applications.

The occurrence of suboptimal lesions (defined as ΔImpedance ≤ 5% or total energy ≤330 J) was particularly common (10.3%) in applications outside of the 16 g window. However, a narrower CF window (6–18 g) may be needed for ablation of areas with potentially thick atrial walls. In our analysis, a higher number of applications with low cumulative applied energy was observed in these PV segments, even when lower CF values were applied (8.4% of applications with CF > 18 g, Phi coefficient 0.093, *p* = .003). Further research is required to assess the effect of a CF window on procedural success as well as on clinical outcomes after PVI.

Our explorative analysis had some limitations. Applications were exclusively performed using the QDOT Micro catheter (Biosense Webster), as it is the only widely available vHPSD‐dedicated catheter offering optimized temperature monitoring and irrigation at the time of this study. The rates of first‐pass isolation and acute PV reconnection, as well as the need for additional AI‐guided ablation, were not routinely documented. However, the rates of impedance drop can also adequately characterize ablation lesions, as described above. Changes in generator impedance were measured by the system, as the QDOT Micro catheter is unable to detect changes in local impedance. Moreover, further research is needed to investigate the role of temperature‐guided downregulation of energy on procedural and clinical outcomes. Additionally, the role of pre‐existing LA wall fibrosis on successful lesion generation in vHPSD ablation is currently unknown. Furthermore, in the majority of applications, nonsteerable sheaths were used. Steerable sheaths can improve catheter stability and hence positively impact lesion creation.^31^

## 5 CONCLUSION

We described the optimal contact‐force profile for a sufficient lesion generation using the QDOT Micro catheter in vHPSD Mode (90 W, 4 s), demonstrating that applications with a mean CF <6 g and > 22 g were associated with an inadequate impedance drop and total applied energy, respectively. Applications outside the optimal CF window may be responsible for inappropriate lesion formation, particularly at superior parts of the PVs with thick atrial walls, thus potentially providing the basis for electrical PV recovery.

## AUTHOR CONTRIBUTIONS

Concept/design: Ukena, Rostock, Spittler, Böhm, Mahfoud. Data Analysis/Statistics: Bismpos, Wintrich, Pavlicek, Benz, Ferro, Spittler, Rostock, Ukena. Drafting article: Bismpos, Ukena. Critical revision of article: Ukena, Rostock, Mahfoud, Böhm.

## CONFLICT OF INTEREST STATEMENT

J.W. is supported by the Deutsche Forschungsgemeinschaft (Sonderforschungsbereich (SFB)TTR 219, S‐01) and the Deutsche Herzstiftung. M.B. is supported by the Deutsche Forschungsgemeinschaft (German Research Foundation; SFB TTR 219, project number 322900939) and reports personal fees from Abbott, Amgen, AstraZeneca, Bayer, Boehringer Ingelheim, Cytokinetics, Edwards, Medtronic, Novartis, Recor, Servier, and Vifor during the conduct of the study. F.M. is supported by Deutsche Gesellschaft für Kardiologie (DGK), Deutsche Forschungsgemeinschaft (SFB TRR219, Project‐ID 322900939), and Deutsche Herzstiftung. He has received speaker honoraria/consulting fees from Ablative Solutions, Amgen, AstraZeneca, Bayer, Boehringer Ingelheim, Inari, Medtronic, Merck, ReCor Medical, Servier, and Terumo. C.U. has received speaker honoraria/ consulting fees from Aurigen, Bayer, Biosense Webster, Bristol Myers Squibb, Medtronic, Pfizer, and Recor Medical. Their institution (Saarland University) has received scientific support from Ablative Solutions, Medtronic, and ReCor Medical. T.R. has received consulting fees/honoraria from Boehringer Ingelheim, Bayer, St. Jude Medical, and has received a research grant from Boehringer Ingelheim.

## DECLARATIONS

Approval of the research protocol: yes.

Informed consent: yes.

## Supporting information


Table S1

Figure S1

Figure S2

Figure S3
