# Supplementary material for: The “16‐gram window” of contact‐force: A new criterion for very high‐power short‐duration ablation
Source: J Arrhythm. 2025 May 6;41(3):e70076. doi: 10.1002/joa3.70076 (PMC12053087; doi:10.1002/joa3.70076)
Supplement: Supplementary file 1 — Table S1 [file JOA3-41-e70076-s003.docx]

**Supplement**

**Supplement Table S1 – Ablation metrics related to impedance drop and total applied energy**

|  | ΔImpedance ≤5%  (n=151) | ΔImpedance >5%  (n=3730) | p | Total energy ≤330J (n=117) | Total energy >330J  (n=3764) | p |
| --- | --- | --- | --- | --- | --- | --- |
| Duration (ms) | 3987±60 | 3982±33 | 0.143 | 3936±108 | 3984±28 | <0.001 |
| Maximum temperature (°C) | 46.4±4.3 | 48.9±3.9 | <0.001 | 55.5±6.2 | 48.6±3.7 | <0.001 |
| Temperature rise (°C) | 11±4.2 | 14.1±3.8 | <0.001 | 20±6 | 13.8±3.6 | <0.001 |
| Baseline impedance (Ohm) | 114.6±10.6 | 118.1±10.5 | <0.001 | 114.9±13.2 | 118±10.5 | 0.002 |
| ΔImpedance | 4.5±0.8 | 10.3±2.9 | <0.001 | 11.6±4 | 10±3 | <0.001 |
| Mean contact force (g) | 9.9±6.5 | 12.9±6.5 | <0.001 | 17.6±9 | 12.6±6.4 | <0.001 |
| Minimum contact force (g) | 3.5±3.3 | 5.8±4.6 | <0.001 | 8.2±6.1 | 5.6±4.5 | <0.001 |
| Maximum contact force (g) | 19±12.5 | 23,7±12.4 | <0.001 | 32.4±18.6 | 23.3±12.2 | <0.001 |
| Contact force variability (g) | 15.4±12 | 17.9±11 | 0.007 | 24.3±16.8 | 17.6±10.7 | <0.001 |
| CFvar60% (g) | 6.2±6.4 | 7±4.9 | 0.43 | 9.5±8 | 6.9±4.8 | <0.001 |
| CFvar60% >10g | 14.7% | 18,4% | 0.281 | 34.2% | 17.8% | <0.001 |
| Intermittent loss of contact | 22.7% | 8.3% | <0.001 | 1.8% | 9.1% | 0.002 |
| Total applied energy (J) | 333±16 | 334±8 | 0.131 | 308±40 | 335±1 | <0.001 |

Values are mean±standard deviation or numbers (percentage).

**Supplement Table S2 – Ablation metrics related to right and left pulmonary veins**

|  | Total  (n=3881) | Left PV’s  (n=1914) | Right PV’s  (n=1799) | Non-PV lesions  (n=168) | p-value |
| --- | --- | --- | --- | --- | --- |
| Duration (ms) | 3982.9±34.3 | 3982.5±33.8 | 3983±30.5 | 3987.3±64.4 | 0.219 |
| Maximum temperature (°C) | 48.8±4 | 48.9±3.9 | 48.6±4 | 50±4 | <0.001 |
| Temperature rise (°C) | 14±3.8 | 14.1±3.7 | 13.8±3.9 | 14.8±4.1 | 0.002 |
| Baseline impedance (Ohm) | 117.9±10.6 | 118.4±11 | 117.8±10.1 | 114.2±9.5 | <0.001 |
| ΔImpedance (%) | 10.1±3.1 | 10.6±3.2 | 9.6±2.8 | 9.2±2.7 | <0.001 |
| ΔImpedance ≤5% | 150 (3.9%) | 55 (2.9%) | 78 (4.3%) | 17 (10.1%) | <0.001 |
| Mean contact force (g) | 12.8±6.6 | 12.2±6.2 | 13.3±6.7 | 14.6±7.6 | <0.001 |
| Minimum contact force (g) | 5.7±4.6 | 5.7±4.3 | 5.6±4.8 | 7.2±4.9 | <0.001 |
| Maximum contact force (g) | 23.6±12.5 | 22±12 | 25±12.5 | 25.9±14.5 | <0.001 |
| Contact force variability (g) | 17.8±11 | 16.3±10.4 | 19.4±11.2 | 18.7±12.5 | <0.001 |
| CFvar60% (g) | 7±4.9 | 6.3±4.6 | 7.7±5.1 | 7.3±5.1 | <0.001 |
| CFvar60% >10g | 703 (18.2%) | 250 (13.1%) | 413 (23.2%) | 40 (23.8%) | <0.001 |
| Intermittent loss of contact | 341 (8.8%) | 144 (7.6%) | 182 (10.2%) | 15 (8.9%) | <0.001 |
| Total energy (J) | 334.3±8.4 | 334.3±5.7 | 334.4±9.2 | 331.9±18.3 | <0.001 |
| Total energy <330J | 117 (3%) | 69 (3.6%) | 38 (2.1%) | 10 (6%) | 0.002 |

Values are mean±standard deviation or numbers (percentage).

**Supplement Figure S1 - Rates of suboptimal vHPSD applications related to deciles of mean contact force**

**
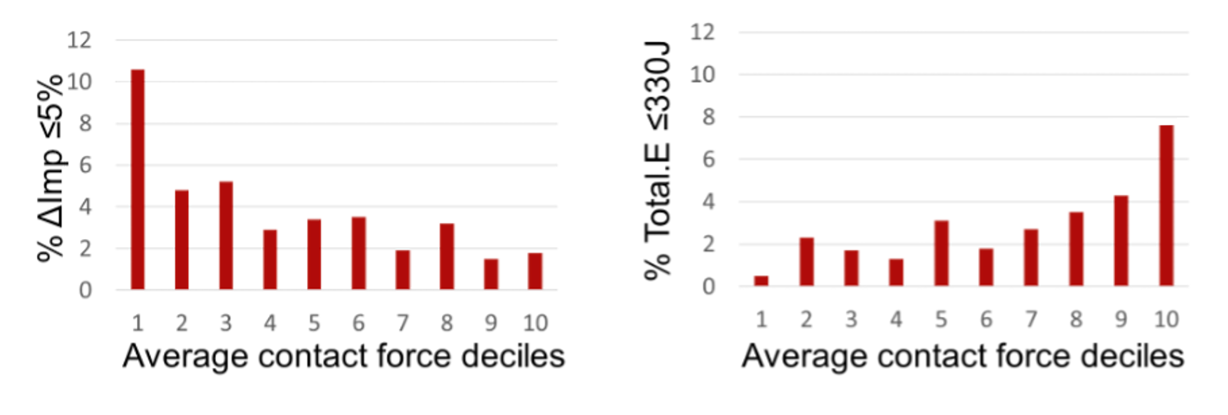
**

**Supplement Figure S2A and S2B -** **Rates of suboptimal vHPSD applications related to the first and last decile of mean contact force**


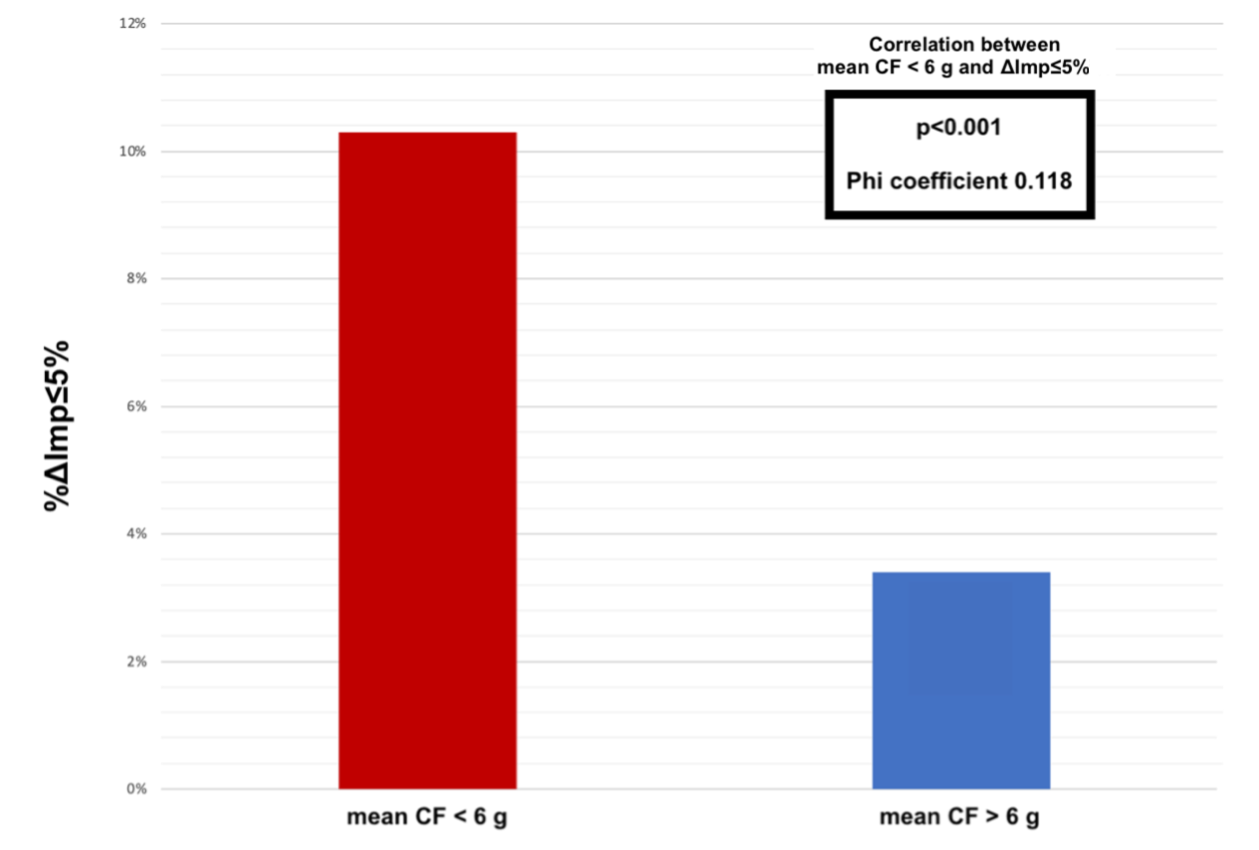


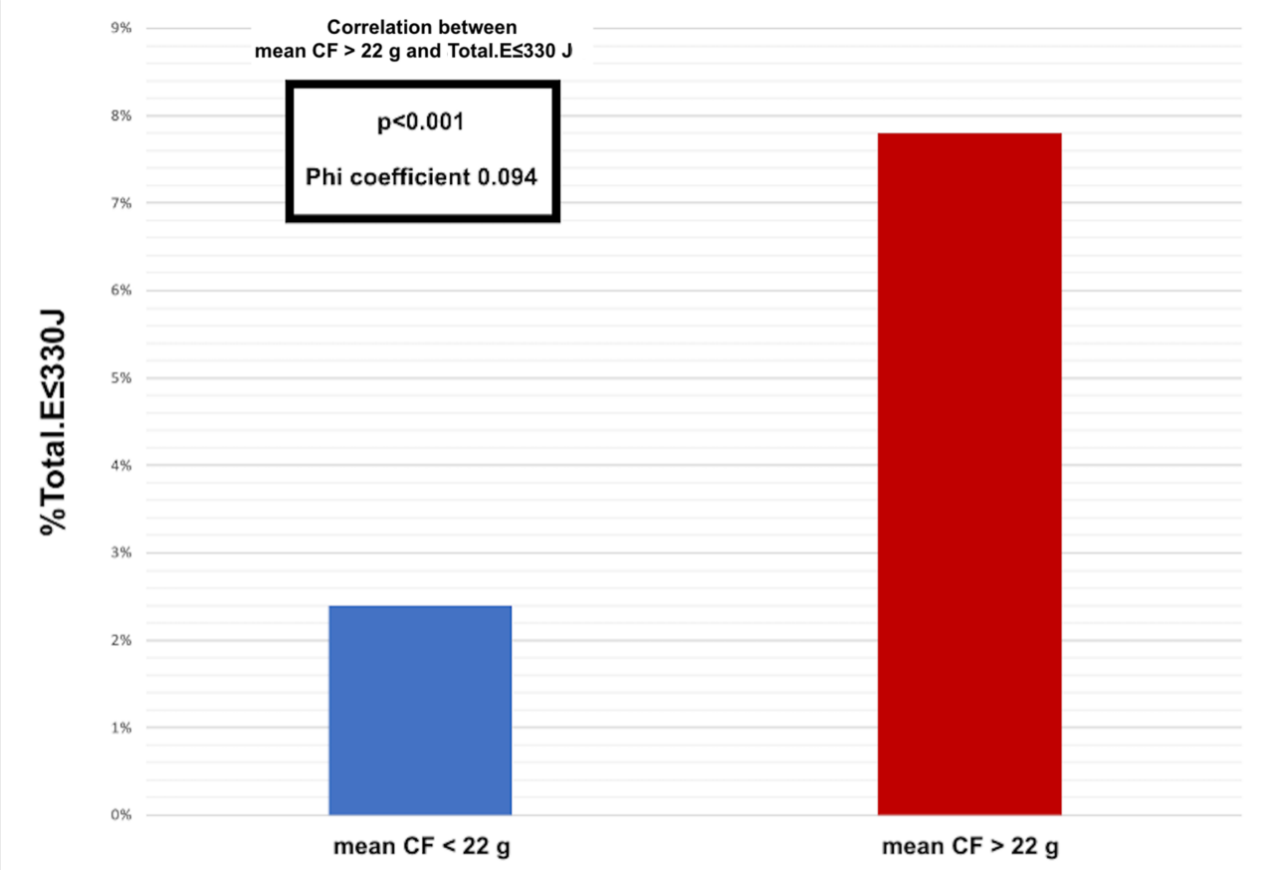


**Supplement Figure S3A and S3B -** **Localized rates of suboptimal vHPSD applications related to the first and eight decile of mean contact force**


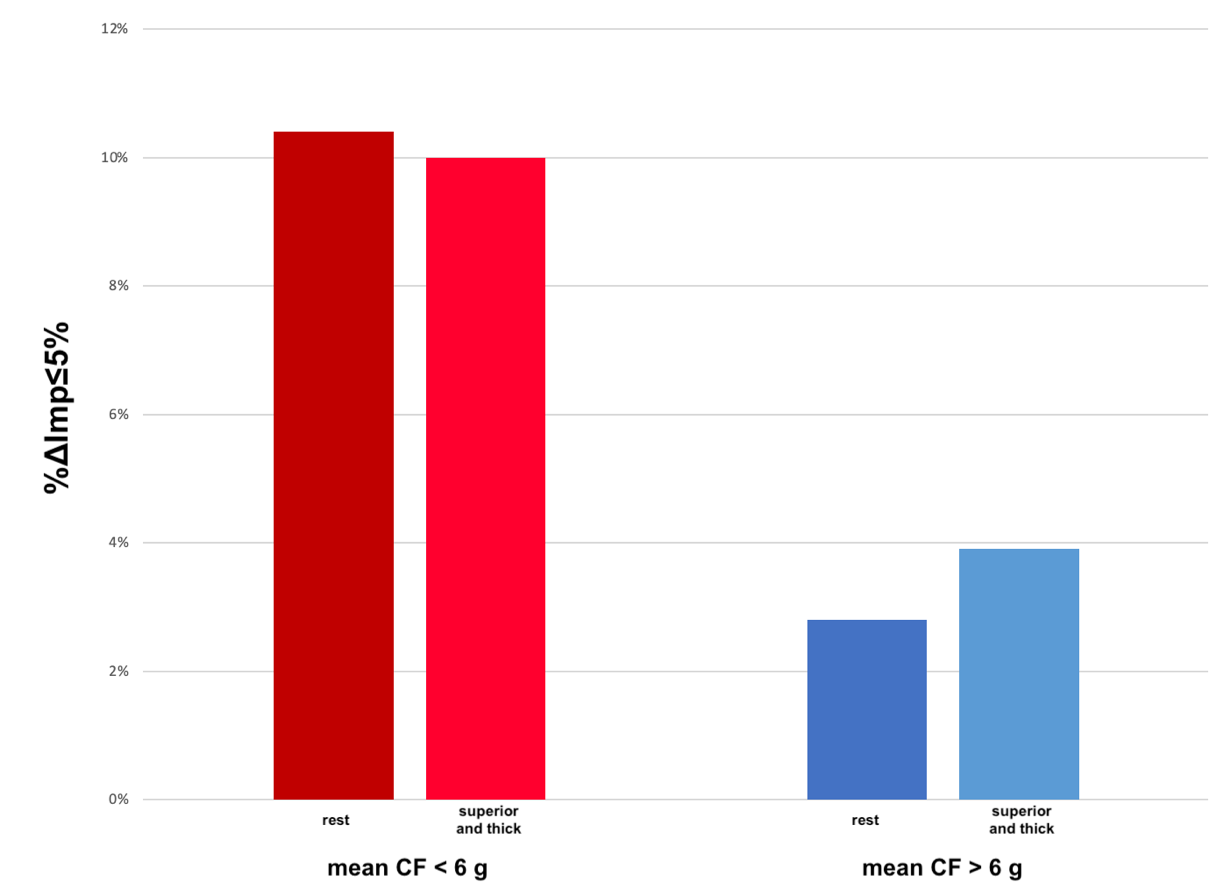


**Supplement Table S1. Ablation metrics related to impedance drop and total energy.** Normally-distributed values are reported as mean±standard deviation (SD), non-normally distributed values are reported as median and Q1-Q3. Categorial values are reported as n (%). Abbreviations: CFvar60%=significant contact force variability; ΔImpedance=impedance drop; g=grams; J=Joule; ms=milliseconds; °C=grads Celsius

**Supplement Table S2. Ablation metrics related to right and left pulmonary veins**

Normally-distributed values are reported as mean±standard deviation (SD), non-normally distributed values are reported as median and Q1-Q3. Categorial values are reported as n (%). Abbreviations: CFvar60%=significant contact force variability; ΔImpedance=impedance drop; g=grams; J=Joule; ms=milliseconds; PVs=pulmonary veins; °C=grads Celsius

**Supplement Figure S1. Rates of suboptimal vHPSD applications related to deciles of mean contact force**

Abbreviations: ΔImp=impedance drop; J=Joule; Total.E=total applied energy; vHPSD=very high power short duration

**Supplement Figure S2A and S2B.** **Rates of suboptimal vHPSD applications related to the first and last decile of mean contact force**

Abbreviations: ΔImp=impedance drop; J=Joule; Total.E=total applied energy; vHPSD=very high power short duration

**Supplement Figure S3A and S3B.** **Localized rates of suboptimal vHPSD applications related to the first and eight decile of mean contact force**

Abbreviations: ΔImp=impedance drop; J=Joule; Total.E=total applied energy; vHPSD=very high power short duration
