# Supplementary figures and images for: The “16‐gram window” of contact‐force: A new criterion for very high‐power short‐duration ablation
Source: J Arrhythm. 2025 May 6;41(3):e70076. doi: 10.1002/joa3.70076 (PMC12053087; doi:10.1002/joa3.70076)

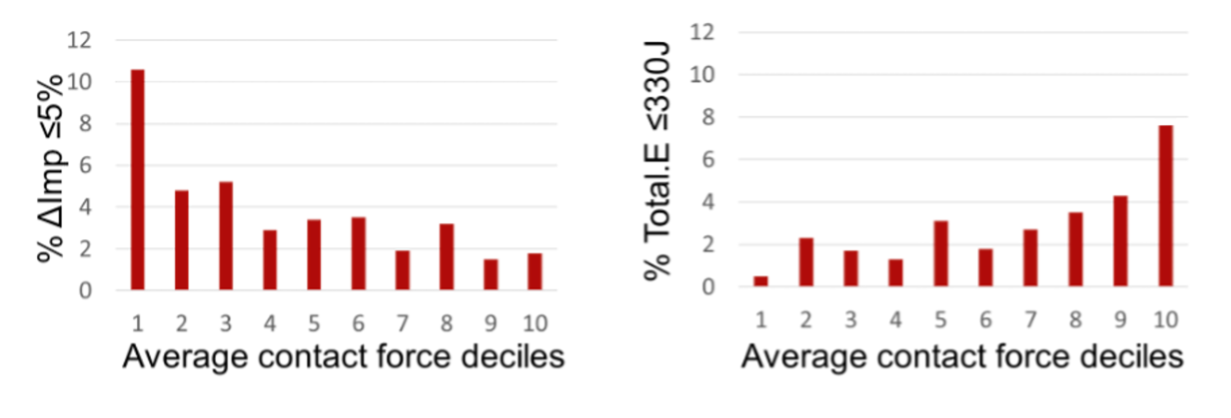

Supplement: Supplementary file 2 — Figure S1 [file JOA3-41-e70076-s002.png]

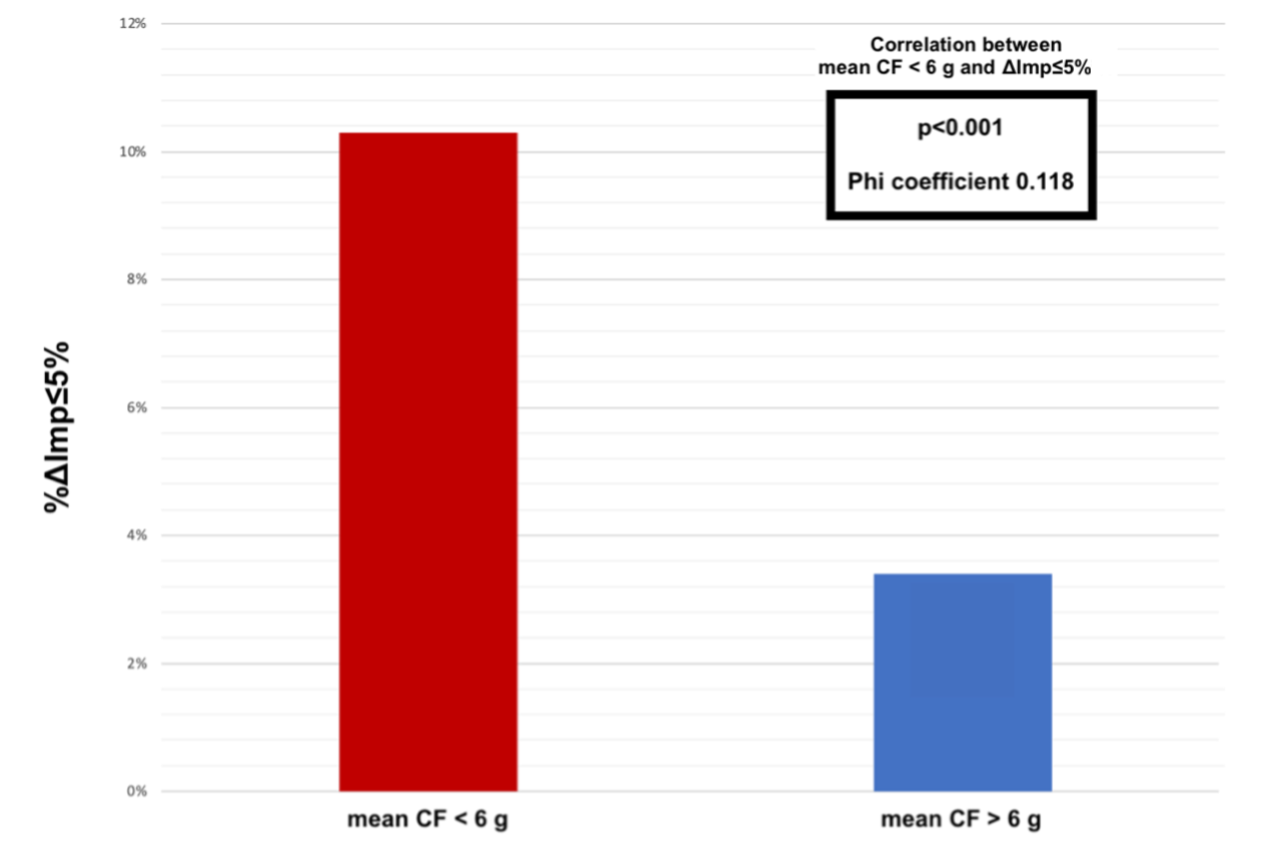

Supplement: Supplementary file 3 — Figure S2 [file JOA3-41-e70076-s004.zip › JOA3_70076_f2_Figure S2A.png]

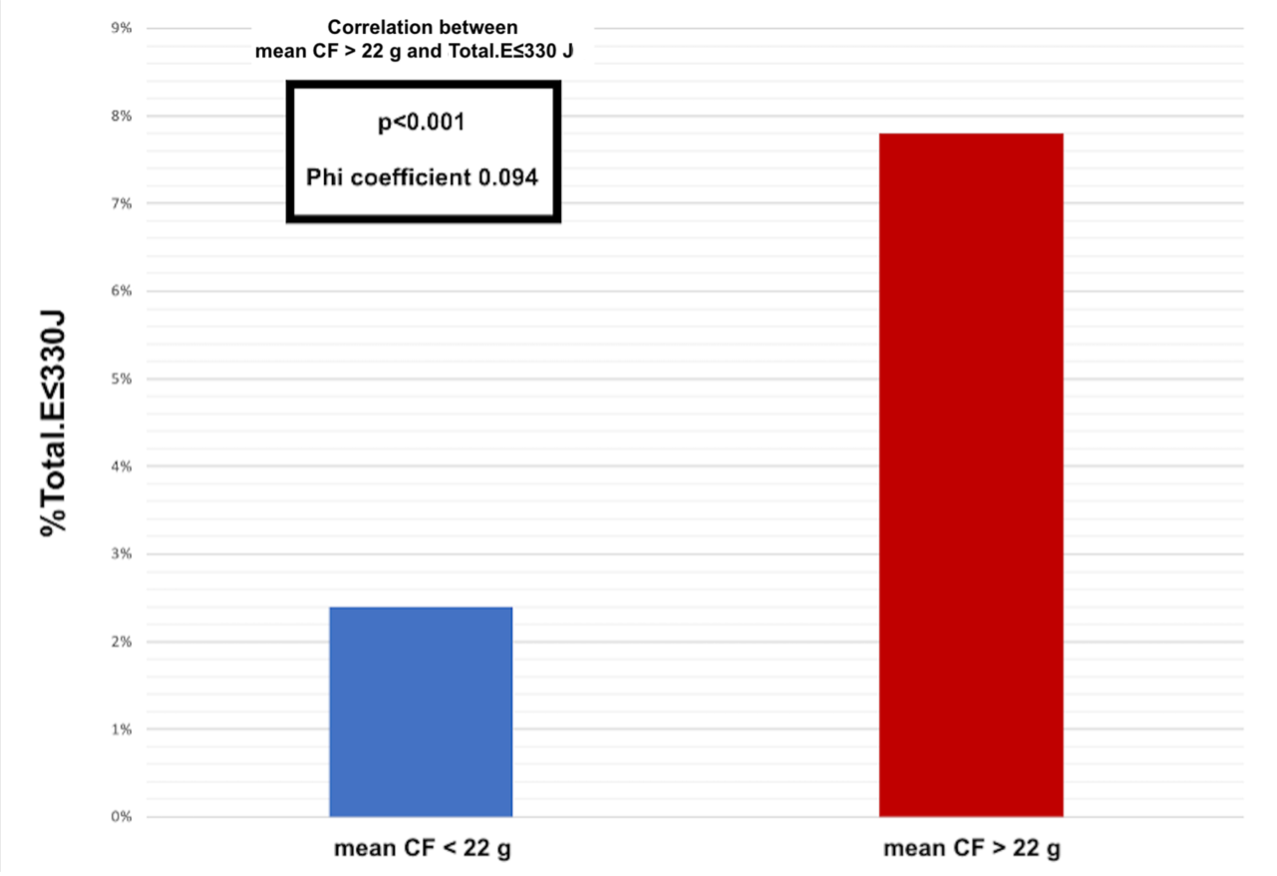

Supplement: Supplementary file 3 — Figure S2 [file JOA3-41-e70076-s004.zip › JOA3_70076_f2_Figure S2B.png]

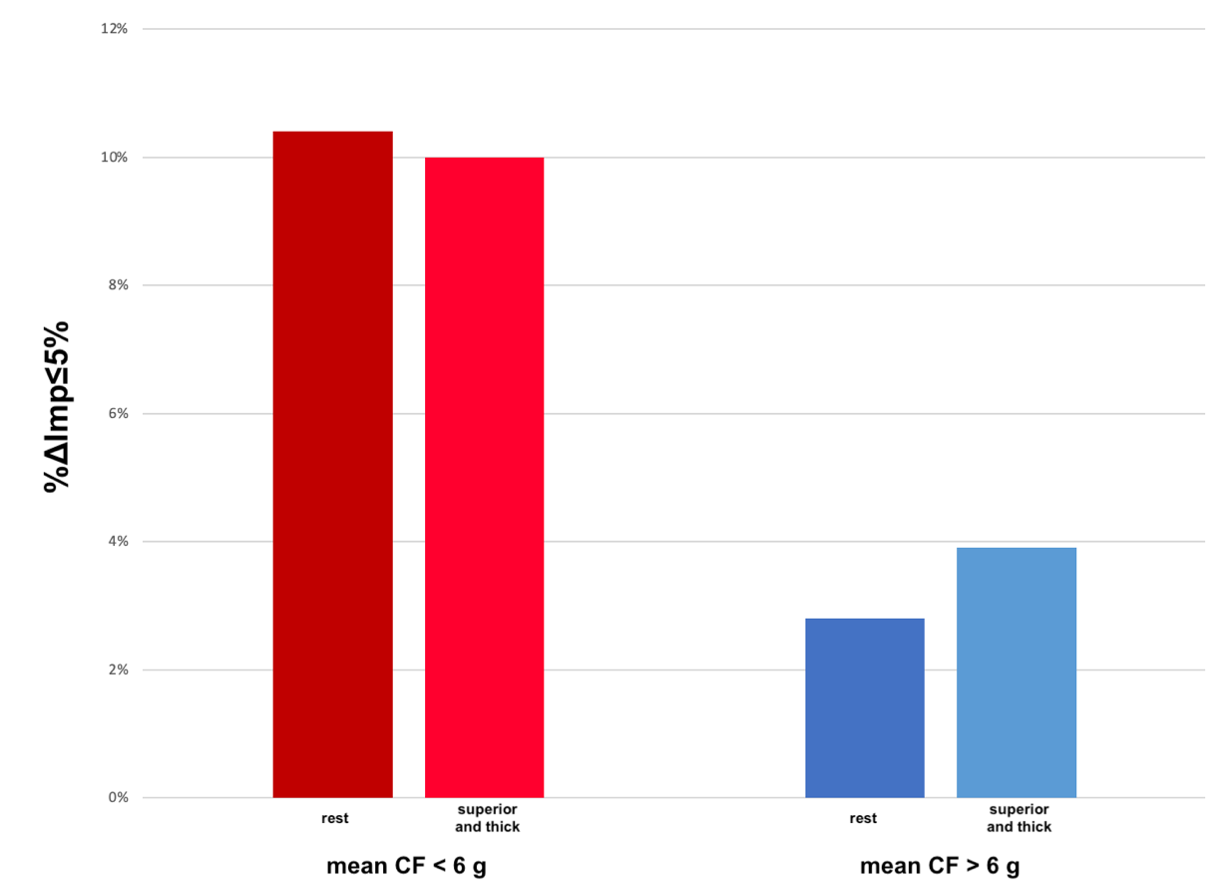

Supplement: Supplementary file 4 — Figure S3 [file JOA3-41-e70076-s001.zip › JOA3_70076_f3_Figure S3A.png]

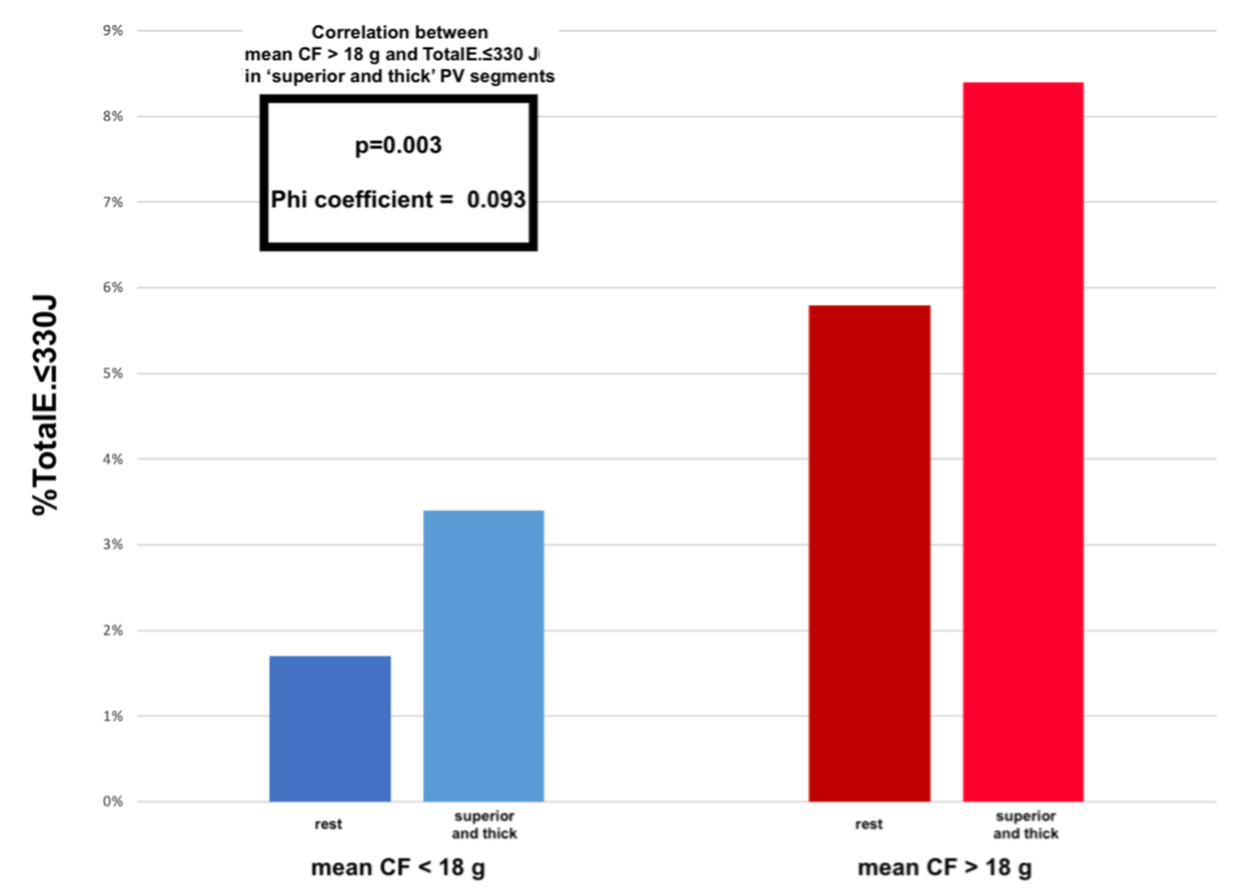

Supplement: Supplementary file 4 — Figure S3 [file JOA3-41-e70076-s001.zip › JOA3_70076_f3_Figure S3B.png]
